# Calcein-effluxing human colon cancer cells are enriched for self-renewal capacity and depend on β-catenin

**DOI:** 10.18632/oncotarget.883

**Published:** 2013-02-18

**Authors:** Joshua E. Allen, Wafik S. El-Deiry

**Affiliations:** ^1^ Laboratory of Translational Oncology and Experimental Cancer Therapeutics, Department of Medicine (Hematology/Oncology), Penn State Hershey Cancer Institute; ^2^ American Cancer Society, Atlanta, Georgia

**Keywords:** cancer stem cell, beta-catenin, Wnt, side population, calcein, CloP, colon cancer

## Abstract

Putative cancer stem cells are a subpopulation of cancer cells that give rise to chemotherapy resistance and are therefore of prognostic and therapeutic interest, though their identification remains elusive in colon cancer due to lack of reliable and accurate markers. We previously identified a p53-dependent putative cancer stem cell population, the calcein low population (C^lo^P), based on their exclusive efflux of the fluorescent dye Calcein. This functional identification method enables comparative live cell studies of subpopulations without differential toxicity that occurs with traditional Hoechst methods, which has confounded conclusions and limited the utility of this cancer stem cell marker. In this study, we examined the cancer stem cell-like properties of the C^lo^P population *in vivo* in comparison with the parental and calcein-high population (C^hi^P) in human colon cancer xenografts. Serial dilution xenograft experiments in NOD/SCID mice revealed that the C^lo^P is only marginally more tumorigenic compared to the C^hi^P or parental cells. However, serial passage of these tumors revealed that the C^lo^P is uniquely enriched for self-renewal capacity *in vivo* compared to the other populations. Immunohistochemical analysis of these tumors revealed that the C^lo^P possesses increased levels of nuclear β-catenin and furthermore, siRNA-mediated knockdown of β-catenin significantly reduced the C^lo^P population. These findings highlight the C^lo^P as an important subpopulation of tumor cells that are exclusively endowed with the ability to self-renew and propagate tumors. The dependency of the C^lo^P on β-catenin provides a molecular explanation for this ability and suggests that this population can and should be therapeutically targeted by inhibition of Wnt signaling.

## INTRODUCTION

The heterogeneity of tumors has become increasingly apparent along with the emerging importance of the tumor microenvironment. Among the various subpopulations of cells that comprise the tumor, cancer stem cells (CSCs) are a unique subset of tumors cells that are exclusively responsible for potentiating and propagating tumors. However, the CSC literature in some disease settings such as colon cancer is shrouded in controversies regarding how to best define or identify CSCs and even the veracity of their existence altogether.

CD133^+^ as a colon cancer CSC marker has been debated, as some reports have shown equivalent tumorigenic potential between CD133^+^ and CD133^−^ subpopulations [1]. Other approaches have focused on functional assays for CSC identification. In colon cancer, such methods include enhanced activity of the enzyme aldehyde dehydrogenase 1 (ALDH1) amongst CSCs [2, 3] or the increased efflux of small molecules by ABC transporters [4]. The latter property is often identified by efflux of the DNA-binding dye Hoechst 33342 using flow cytometry analysis to identify the side population (SP) that does not retain this fluorescent molecule [5, 6]. The SP has been identified in numerous tumor types and directly linked to therapy resistance due to its efflux of several chemotherapies.

While the SP clearly confers multidrug-resistance, its exclusive tumorigenic potential is less clear [7, 8]. The validity of studies comparing the tumorigenicity of the SP and non-SP has been questioned as the dye used in these identification assays for SP is Hoechst 33342, which is a DNA-intercalating molecule that we and others have reported to induce p53 and cause cell death [9-12]. This has important implications for comparative studies as cells that do not retain the dye, i.e. the non-SP, will be more affected by the cytotoxic properties of the dye due to relatively increased retention of Hoechst and this may explain why these cells are less tumorigenic than the SP. This led us to develop a nontoxic alternative CSC identification method that revealed the calcein-low population (C^lo^P) [9]. The C^lo^P is evident in several human cancer cell lines, is p53-dependent, is inhibited by the ABC-transporter inhibitor verapamil, and possesses significant overlap with the SP population as expected [9]. To further evaluate CSC-like properties, we now evaluate the ability of the C^lo^P to initiate and propagate human colon cancer tumors *in vivo* to definitively gauge the significance of this unique population of tumor cells.

## RESULTS

### The CloP are enriched for CD133 expression in some colon cancer cells

We previously demonstrated that the C^lo^P overlaps with the SP [9] but its overlap with other functional or surface markers was not explored. We therefore examined the overlap of the C^lo^P with CD133 and CD26 surface expression in human colon cancer cell lines (Table 1; Figure S1). Profiling these markers in a panel of human colon cancer cell lines revealed heterogeneous expression of CD133 and CD26, though the C^lo^P was consistently in the range of ~1-2% of the total population and. CD133 was highly expressed in RKO and SW620 cells at >60% of the total population. The CD26^+^ population was rare in most tested cell lines with the exception of SW620 (Figure S1). Comparing the overlap of the C^lo^P with these canonical markers did not reveal any striking enrichment for CD133 or CD26 within the C^lo^P except for HT-29 cells, which were highly enriched for CD133^+^ cells.

**Table 1:** The CloP is enriched for CD133 expression in HT-29 but not other colon cancer cell lines

		Cell Line			
		RKO	HT29	HCT116 p53−/−	SW620
	CloP	5.4%	4.0%	4.6%	3.8%
	CloP (Verap.)	3.0%	3.2%	2.3%	1.7%
Whole Population	CD133+	88.1%	9.4%	61.2%	12.3%
	CD26+	2.2%	1.1%	1.2%	26.7%
	CD133+CD26+	2.2%	1.1%	1.2%	8.6%
CloP	CD133+	85.8%	41.4%	68.3%	3.2%
	CD26+	11.1%	1.3%	3.9%	9.8%
	CD133+CD26+	11.1%	1.3%	3.9%	1.0%
Chip	CD133+ enrichment ratio (CloP/Whole)	0.97	4.4	1.12	0.26
	CD133+	88.4%	7.1%	63.1%	6.0%
	CD26+	2.4%	0.7%	0.7%	20.3%
	CD133+CD26+	2.4%	0.7%	0.7%	2.1%

### The CloP is similarly tumorigenic to other populations but is enriched in self-renewal capacity

**Figure 1:**
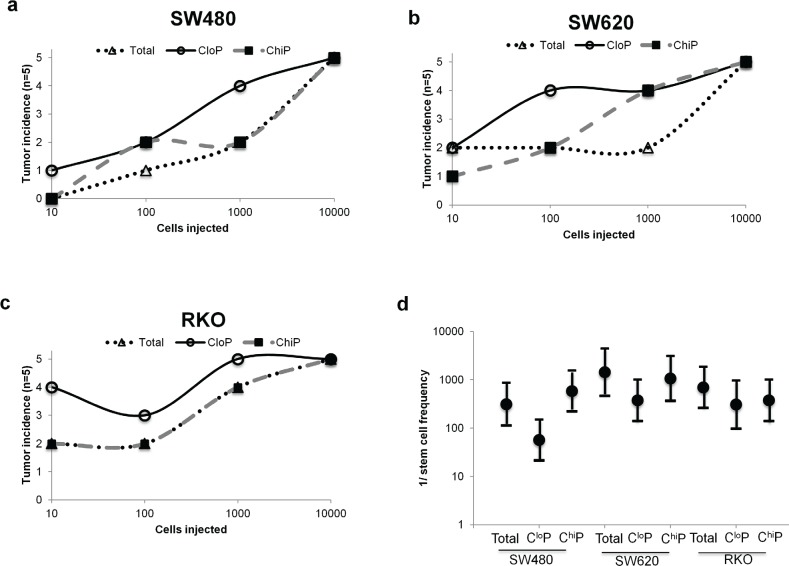
The C^lo^P is similarly tumorigenic to other C^hi^P and parental populations in colon cancer xenografts Sorted C^lo^P, C^hi^P, and parental populations from culture of (a) SW480, (b) SW620, and (c) RKO cancer cell lines injected into the rear flanks of NOD/SCID mice (n=5). Exemplary post-sort purity given in Figure S2. (d) Stem cell frequencies were extrapolated from data in (a-c). Dots represent the estimated stem cell frequency and errors bar represent the upper and lower estimates from the analysis.

We performed serial dilution xenotransplantation of the total population, C^lo^P, and C^hi^P into NOD/SCID mice to determine and compare their tumorigenic potential. In the evaluated cancer cell lines, the C^lo^P possessed a marginally increased number of tumors resulting from injections of <1 000 cells in the majority of xenografts (Figure 1). Extrapolating the stem cell frequency of these different populations for each cell line revealed that the C^lo^P possesses a modestly higher estimated stem cell frequency compared to the C^hi^P or total population, although the differences did not reach statistical significance (Figure 1d). While the C^lo^P appears slightly more tumorigenic in these experiments, these results were not striking and prompted us to further examine differences in CSC-like properties amongst these populations.

**Figure 2:**
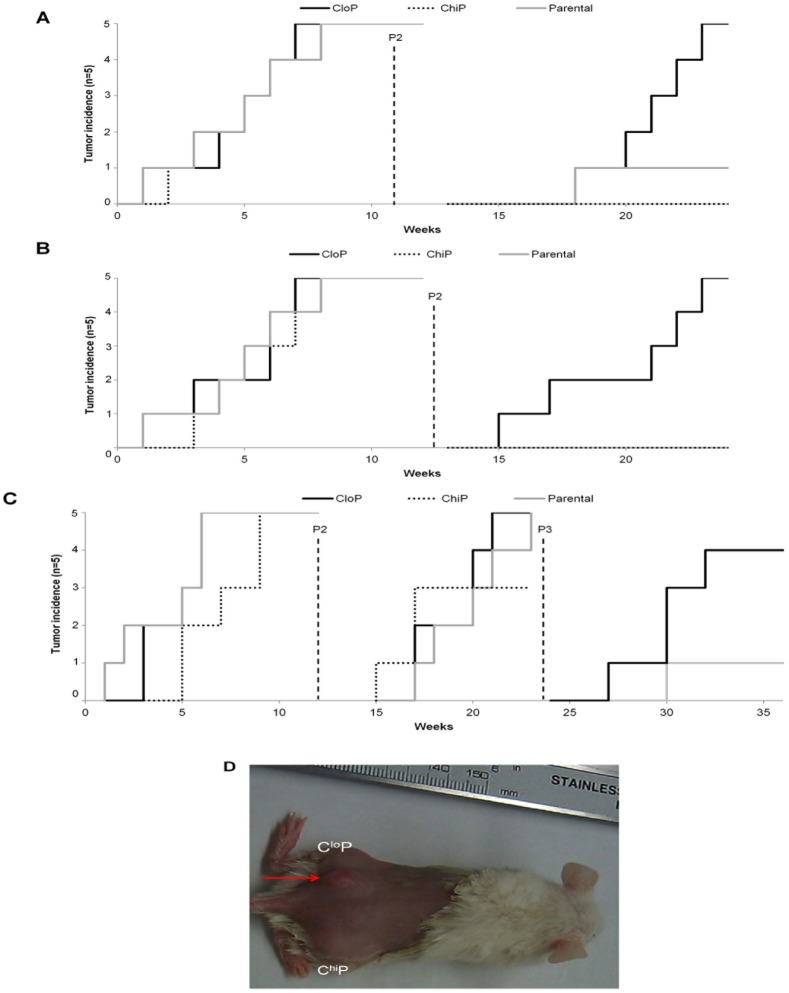
The CloP is exclusively enriched in self-renewal capacity in colon cancer xenografts (a) Tumor incidence with serial passage of SW620 tumor xenografts. (b) Exemplary depilated mouse at 6-weeks following the second passage. Red arrow indicates sight of tumor incidence. (c) SW480 and (d) RKO serial passage experiments as described for SW620 in (a).

We next evaluated the ability of the total, C^lo^P, and C^hi^P population to self-renew using serial passage transplantation of tumors utilizing the same NOD/SCID model as the serial dilution experiments. As in the serial dilution experiments, all populations were capable of initiating tumors with similar kinetics of tumor incidence (Figure 2). However, serial passage of tumors at 12 weeks post-implantation revealed a striking difference in tumorigenic potential amongst the different population in all cell lines. In both the SW480 and SW620 cell lines, exclusively the C^lo^P and not the C^hi^P or total population were capable of reinitiating the tumor at second passage. All populations in the RKO xenografts reinitiated tumors at similar kinetics again upon the second passage. However, only the C^lo^P was capable of tumor formation following the third passage. These results indicate that the total population, C^lo^P, and C^hi^P of human cancer cell lines are capable of initiating tumors but that the C^lo^P possesses the exclusive CSC-like ability to self-renew.

### The CloP is β-catenin-dependent

**Figure 3:**
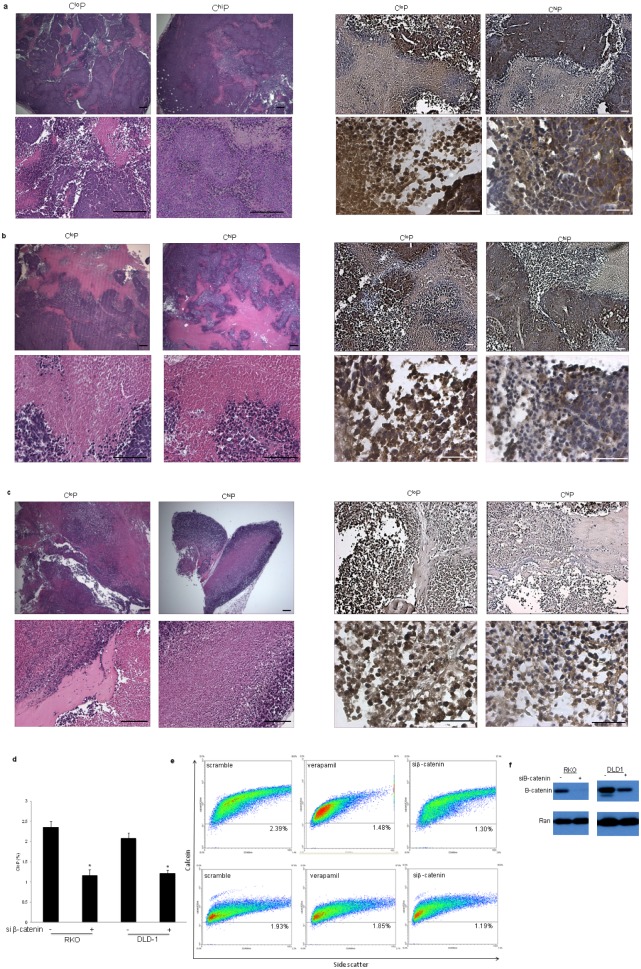
The CloP is β-catenin-dependent Histological and immunohistological analysis of C^lo^P- and C^hi^P-initiated (a) SW620, (b) SW480, and (c) RKO tumor xenografts harvested 12-weeks post-injection of 10,000 cells into NOD/SCID mice as described in Figure 1. (d) Flow cytometry analysis of the C^lo^P following transient knockdown of β-catenin using siRNA at 48 hours post-knockdown with (e) an exemplary C^lo^P scattergram analysis as described Figure 1 (n=3). Error bars indicate standard deviation of replicates. *P<.05 by student's two-tailed t test. (f) Verification of transient knockdown of β-catenin by Western blot analysis at 48 hours post-knockdown.

The unique self-renewal capability of the C^lo^P warranted further investigation to provide a molecular explanation for this exclusive property. We investigated C^lo^P- and C^hi^P-initiated tumor xenografts for expression levels and localization of pAkt, Gli-1, and β-catenin as markers of signaling pathways that are directly involved in self-renewal. We found that tumors generated by both populations had largely necrotic central regions, which are observed in human tumors, surrounded by regions with higher tumor cell density (Figure 3a-c, data not shown). Out of the tested self-renewal signaling pathway markers, we found that β-catenin expression levels and nuclear localization was apparently elevated in C^lo^P-initiated tumors whereas C^hi^P-initiated tumors generally contained lower intensity and more diffuse staining for β-catenin. These results highlight the heterogeneity of β-catenin localization amongst tumor subpopulations generated from the same isogenic background. Despite the nearly ubiquitous activating mutations in the Wnt signaling pathway seen in human colon cancer, similar observations of β-catenin expression heterogeneity have been reported for primary patient samples [13, 14].

We investigated the dependency of the C^lo^P on β-catenin. siRNA-mediated knockdown of β-catenin significantly reduced the C^lo^P in the RKO and DLD-1 cell lines, though to a lesser extent in DLD-1 potentially due to a less robust knockdown of β-catenin (Figure 3d-f). These data demonstrate that C^lo^P-initiated tumors contain elevated nuclear β-catenin, which is important for the maintenance of this unique population of tumor cells.

## DISCUSSION

CD133^+^ is a previously described marker of CSCs in human colon cancer, though it has been contested, and within this subpopulation CD26^+^ was described as a marker of human colon cancer CSCs with exclusive metastatic potential [15]. Our studies found that CD133 was highly expressed in several colon cancer cell lines, which is an unexpectedly high expression for a CSC marker that has been previously noted [16-18]. In contrast, the C^lo^P was consistently in the range of ~1-2% of the total population, which agrees with our previous observations [9]. We also found that CD26^+^ population in the examined colon cancer cell lines was unappreciable except SW620 cells, which is expected as this is the only metastatic colon cancer cell line in the tested panel [15]. Comparing the overlap of the C^lo^P with these canonical markers did not reveal any striking enrichment for CD133 or CD26 within the C^lo^P except for HT-29 cells, which were highly enriched for CD133^+^ cells. The heterogeneity of these results and the unreliable nature of surface markers in human colon cancer CSC studies prompted us to examine the ability of the C^lo^P to initiate tumors *in vivo* and the exclusivity of this ability as a more direct and pertinent assay.

Our observations and others clearly define β-catenin as potential drug target for depleting putative CSCs that drive tumor progression [19]. This in agreement with recent reports by others and us that intimately link β-catenin to the integrity of the CSC population, particularly with respect to self-renewal [13, 16]. Nuclear β-catenin is evident in 60% of resected colon cancers, though its intratumoral distribution of nuclear expression is heterogeneous and associated with a poor prognosis [20, 21]. Wnt signaling has been intimately linked to the CSC population, including regulation of CSC-associated genes such as CD133, CD44, and MDR1 [22-24]. Colon cancer cells with increased Wnt signaling were shown to possess increased tumorigenic potential and CSC properties in vitro [13]. However, a more recent study refuted this observation and suggested a positive crosstalk between Wnt and MAPK signaling. Our studies suggest that the C^lo^P, which potentiates tumors with increased nuclear β-catenin, have similar initial tumorigenic potential but instead possess a unique ability to continually propagate tumors.

While targeting the Wnt signaling pathway has proven difficult in the past, new therapies are in discovery and early clinical trials phases that target Wnt ligands or downstream mediators and effectors of this pathway [25]. These therapeutics provide an exciting opportunity to attack tumors by targeting CSCs, which is a promising therapeutic and has been suggested with other potential targets such as p53 restoration [9, 26] or inhibiting the insulin-like growth factor-1 receptor (IGF1R) [16] that have been suggested by us and others. Future studies should examine the potential of such therapeutics to deplete the C^lo^P and their affect on overall tumor progression and should also address the importance of Notch, GSK3-β, FoxoM1, and miR-371-373 that can regulate β-catenin activity. It will be important to examine the contribution of the C^lo^P to the CTC population, which is of great clinical importance in colon cancer [27] and may be related to the CSC population [28, 29]. Ultimately, the prevalence and prognostic value of the C^lo^P should be evaluated in primary tumor specimens to further substantiate the clinical significance of these cells that we demonstrate preclinically herein.

## MATERIALS AND METHODS

### Cell culture

Cell lines were obtained from ATCC and cultured under the recommended conditions. For expression analysis of CD133, CD26, and calcein-efflux in cultured cells from culture under resting conditions and log-phase growth were analyzed as previously described by flow cytometry analysis [9] using Calcein AM (Molecular Probes, Junction City, OR) at a working concentration 1 nM for 30 minutes with vortexing. Gating was performed with isotype controls and data was processed with FlowJo software. Exemplary gating, controls, scatter plots, and histograms are shown in Figure S1. Antibodies and siRNA for β-catenin were obtained from Cell Signaling. Western blot analysis and transfection of siRNA was performed as previously described [30].

### *In vivo* studies

All animal experiments were approved by the Institutional Animal Care and Use Committee at Penn State Hershey Medical Center. 8-week-old female NOD/SCID mice were obtained from Charles River. Subcutaneous xenografts were established by injection of tumor cells in a 200μL suspension of 1:1 PBS:Matrigel (BD) in rear flanks. Cells were maintained on ice following sorting and injected at equivalent numbers of viable cells using serial dilution of stock solutions that were enumerated in triplicate using a Cellometer (Nexcelom Bioscience LLC, Lawrence, MA) and Trypan Blue Solution (Mediatech). C^lo^P and C^hi^P injections were in the same mice with one population per rear flank to minimize inter-mouse variability. Tumor incidence was monitored weekly by palpitation and caliper measurement of the depilated flanks and confirmed at endpoint by necropsy. Week 0 is defined as the time of tumor cell injection. Stem cell frequencies was determined using Extreme Limiting Dilution Analysis software [31].

For serial passage, tumors were harvested at endpoint immediately following sacrifice and subjected to manual and enzymatic digestion using Collagenase type 3 (Worthington) at 155 units/mL in sterile serum- and antibiotic-free RPMI (Mediatech, Inc, Herndon, VA) for 2 hours with intermittent vortexing. Digested tumor cells for each population were filtered through a 100 μm nylon mesh, pooled, enumerated, and reinjected as described above.

## Supplementary Figures


