# Supplementary material for: Calcein-effluxing human colon cancer cells are enriched for self-renewal capacity and depend on β-catenin
Source: Oncotarget. 2013 Feb 18;4(2):184–91. doi: 10.18632/oncotarget.883 (PMC3712565; doi:10.18632/oncotarget.883)
Supplement: Supplementary file 1 [file oncotarget-04-184-s001.docx]

**SUPPLEMENTARY INFORMATION**

**Calcein-effluxing human colon cancer cells are enriched for self-renewal capacity and depend on β-catenin**

**Running title:** Effluxing cells self-renew and depend on β-catenin

Joshua E. Allen and Wafik S. El-Deiry

**
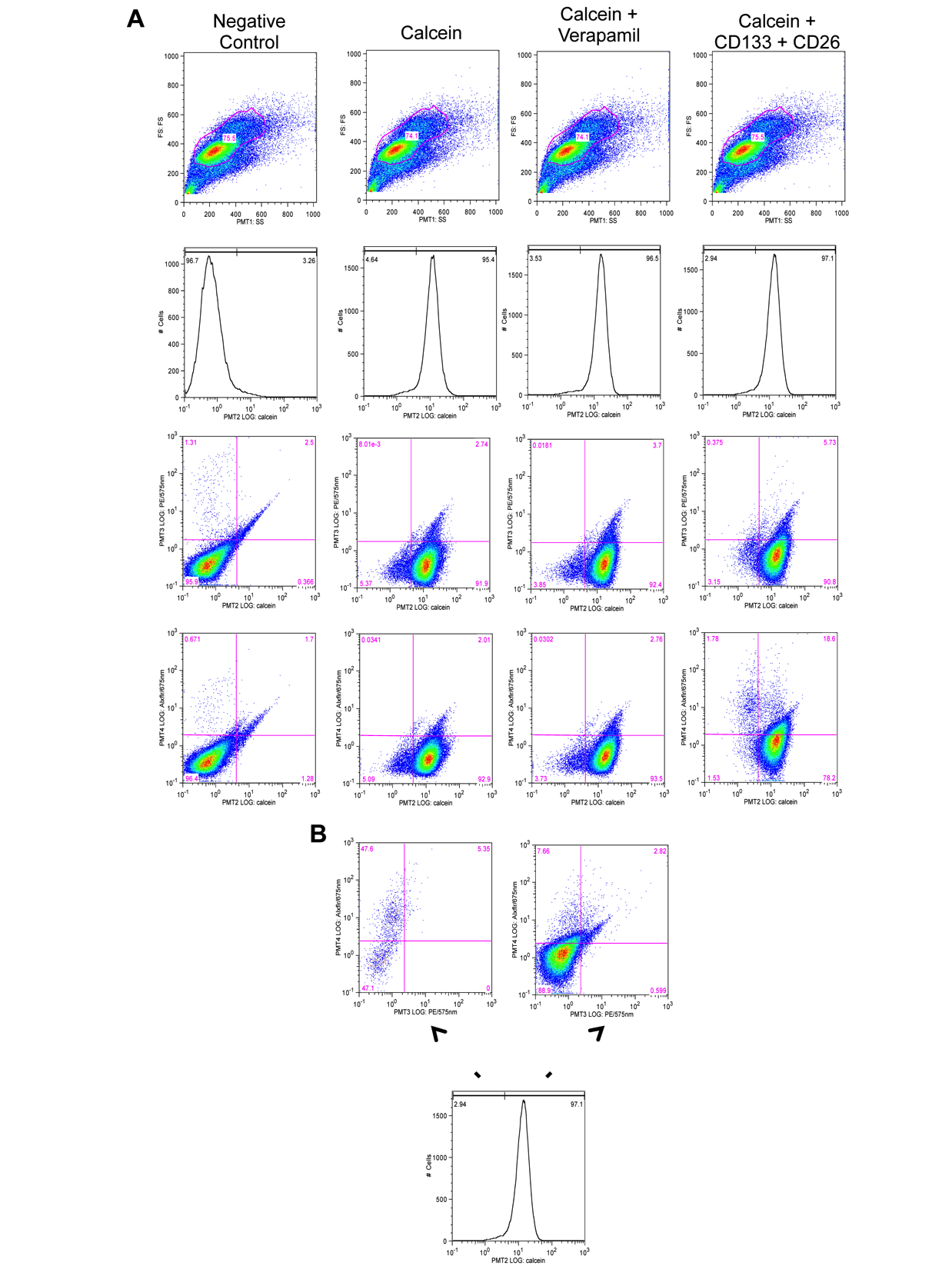
**

**Supplementary Figure S1**

(**A**) Exemplary gating, scatter plots, and histograms for concomitant analysis of CD133, CD26, and calcein populations in HT-29 as described in Table 1. Labels are forward scatter (FS:FS), side scatter (PMT1:SS), calcein (PMT2:calcein), CD133 (PMT4: Alexflr675nm), CD26i (PMT3: PE/575nm). Negative control included incubation with Rabbit and Mouse IgG controls. (**B**) Differential expression of CD133 and CD26 in C^lo^P versus C^hi^P.

**
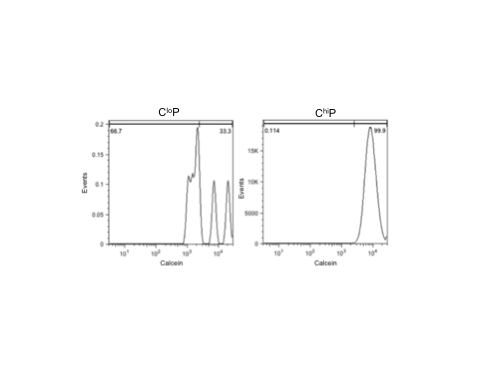
**

**Supplementary Figure S2**

Exemplary post-sort purity for RKO C^lo^P and C^hi^P populations.
